# Absolute Lymphocyte Count Predicts Immune-Related Adverse Events in Patients With Non-Small-Cell Lung Cancer Treated With Nivolumab Monotherapy: A Multicenter Retrospective Study

**DOI:** 10.3389/fonc.2021.618570

**Published:** 2021-05-27

**Authors:** Saeka Egami, Hitoshi Kawazoe, Hironobu Hashimoto, Ryuji Uozumi, Toko Arami, Naomi Sakiyama, Yuichiro Ohe, Hideo Nakada, Tohru Aomori, Shinnosuke Ikemura, Koichi Fukunaga, Masakazu Yamaguchi, Tomonori Nakamura

**Affiliations:** ^1^ Division of Pharmaceutical Care Sciences, Center for Social Pharmacy and Pharmaceutical Care Sciences, Keio University Faculty of Pharmacy, Tokyo, Japan; ^2^ Division of Pharmaceutical Care Sciences, Keio University Graduate School of Pharmaceutical Sciences, Tokyo, Japan; ^3^ Department of Pharmacy, National Cancer Center Hospital, Tokyo, Japan; ^4^ Department of Biomedical Statistics and Bioinformatics, Kyoto University Graduate School of Medicine, Kyoto, Japan; ^5^ Department of Thoracic Oncology, National Cancer Center Hospital, Tokyo, Japan; ^6^ Division of Hospital Pharmacy Science, Keio University Faculty of Pharmacy, Tokyo, Japan; ^7^ Department of Pharmacy, Keio University Hospital, Tokyo, Japan; ^8^ Keio Cancer Center, Keio University School of Medicine, Tokyo, Japan; ^9^ Division of Pulmonary Medicine, Department of Medicine, Keio University School of Medicine, Tokyo, Japan

**Keywords:** absolute lymphocyte count, biomarker, lymphocyte-to-monocyte ratio, neutrophil-to-lymphocyte ratio, nivolumab, non-small-cell lung cancer

## Abstract

**Background:**

Among patients with advanced non-small-cell lung cancer who were treated with nivolumab monotherapy, the association of peripheral blood count data (at baseline and 2 weeks after treatment initiation) with the early onset of immune-related adverse events (irAEs) and treatment efficacy has not been clearly established. This study aimed to identify peripheral blood count data that may be predictive of the development of nivolumab-induced irAEs in a real-world clinical setting.

**Materials and Methods:**

This multicenter observational study retrospectively evaluated consecutive patients with advanced non-small-cell lung cancer undergoing nivolumab monotherapy in the second- or later-line setting between December 2015 and November 2018 at the National Cancer Center Hospital and Keio University Hospital in Japan. The primary endpoint was the association between peripheral blood count data and irAEs during the 6-week study period. Receiver operating characteristic curve and multivariable logistic regression analyses were performed.

**Results:**

Of the 171 patients evaluated, 73 (42.7%) had ≥1 irAE during the first 6 weeks following treatment initiation. The median time to irAEs from the initiation of nivolumab was 15 (interquartile range: 13–28) days. Receiver operating characteristic curve analyses revealed that the optimal cut-off values of the absolute lymphocyte count, neutrophil-to-lymphocyte ratio, and lymphocyte-to-monocyte ratio 2 weeks after treatment initiation for early irAE onset were 820, 4.3, and 2.2, respectively. In multivariable logistic regression analyses, absolute lymphocyte count >820 at 2 weeks after treatment initiation was significantly associated with an increased risk of early onset of any irAE. In contrast, no significant association was observed for the neutrophil-to-lymphocyte ratio (>4.3) or the lymphocyte-to-monocyte ratio (>2.2) at 2 weeks following treatment initiation.

**Conclusions:**

The absolute lymphocyte count >820 at 2 weeks following nivolumab initiation predicts early onset of irAEs during a 6-week study period. Routinely available absolute lymphocyte count, which is measured after the initiation of nivolumab, may be useful for identifying patients at risk of early onset of irAEs.

## Introduction

Lung cancer is a major cause of morbidity and mortality worldwide (1). The development of immune checkpoint inhibitors (ICIs) has markedly modified the treatment paradigm in cancer, leading to durable responses in patients with malignant tumors. Pivotal phase III trials (2–4) have found that ICI monotherapy is markedly superior to standard second-line docetaxel chemotherapy in prolonging survival of previously treated patients with advanced non-small-cell lung cancer (NSCLC). However, ICIs are also associated with the development of immune-related adverse events (irAEs), which remain an unresolved issue in clinical practice (5, 6). Further, Shah et al. (7) reported an association between age and irAEs and identified the risk factors for irAEs in patients treated with ICIs. In contrast, programmed death-ligand 1 (PD-L1) expression was only a predictive biomarker for the efficacy of pembrolizumab. More importantly, routinely available peripheral blood biomarkers predictive of irAEs were inconsistent with those reported in previous studies and thus, remain controversial (8, 9).

In recent years, irAEs induced by the anti-PD-1 antibodies, nivolumab and pembrolizumab, have been shown to be associated with a survival benefit in patients with advanced NSCLC and malignant melanoma (10–19). Early detection of nivolumab-induced irAEs is crucial because of their negative impact on the patient’s quality of life and associated burden on healthcare resources and costs. Routinely available peripheral blood count data, such as the absolute lymphocyte count (ALC), neutrophil-to-lymphocyte ratio (NLR), and lymphocyte-to-monocyte ratio (LMR), have the potential to predict treatment efficacy in patients with advanced NSCLC (20–23). Hence, we hypothesized that peripheral blood count data can also predict nivolumab-induced irAEs in patients with advanced NSCLC. ICIs interrupt immune suppression and activate CD8-positive T lymphocytes in the tumor microenvironment. These activated T lymphocytes not only attack the tumors but also cause irAEs, suggesting that these activated CD8-positive T lymphocytes exert systemic actions (22). Taken together, we explored the hypothesis that an increase in ALC and a related change in blood count ratios may help predict irAEs. To the best of our knowledge, no large-scale multicenter study has investigated the association of peripheral blood count data (at baseline and 2 weeks after treatment initiation) with the early onset of irAEs and treatment efficacy in patients with advanced NSCLC treated with nivolumab monotherapy.

Therefore, this study aimed to identify peripheral blood count data that may be predictive of the development of nivolumab-induced irAEs in a real-world clinical setting.

## Materials and Methods

### Study Design and Patients

This multicenter, retrospective observational study was conducted at the National Cancer Center Hospital, Keio University Hospital, and Keio University Faculty of Pharmacy, Tokyo, Japan. Research members from the Keio University Faculty of Pharmacy acquired data from electronic medical records at the National Cancer Center Hospital and Keio University Hospital. Data integration was performed at Keio University Faculty of Pharmacy, and subsequent statistical analyses were performed at Kyoto University Graduate School of Medicine, Kyoto, Japan. The methodology of this study has been previously reported by our co-author (24).

The subjects were consecutive patients, who were aged ≥20 years, diagnosed with advanced NSCLC, and underwent nivolumab monotherapy in the second- or later-line setting between December 2015 and November 2018 at the National Cancer Center Hospital and Keio University Hospital. Nivolumab was administered at a dose of 3 mg/kg every 2 weeks until August 2018, and thereafter, at a dose of 240 mg/body every 2 weeks according to the prescribing information contained in the package insert. The treatment schedule and follow-up were modified at the clinicians’ discretion according to toxicity profiles. Clinic visits and imaging evaluations were conducted every 6 to 8 weeks starting at treatment initiation according to the Response Evaluation Criteria in Solid Tumors (version 1.1). Patient records were de-identified and analyzed anonymously. The exclusion criteria were as follows (1): history of prior administration of any ICIs and/or investigational drugs as part of a clinical trial or at a previous hospital before the investigation period (2), discontinuation of treatment owing to death or hospital transfer during the first 6 weeks, (3) discontinuation of treatment after the first cycle because of disease progression or adverse events, (4) lack of laboratory data 2 weeks after the first cycle (acceptable range from day 12 to 16), and (5) study participation shorter than 6 weeks (*i.e.*, patients who started treatment between October and November 2018).

### Study Protocol

We used a landmark analysis considering the lead-time bias owing to the time-dependent nature of irAEs (10, 11). Haratani et al. (10) reported that this approach reduced overestimation. Adverse events after the first cycle could be easily detected because the patients were hospitalized. Thus, we focused on the early onset of irAEs and included only patients with controlled disease and those who were alive 6 weeks after treatment initiation. The collected data included patients’ baseline characteristics [age, sex, Eastern Cooperative Oncology Group performance status (scores ranging from 0 to 5, with higher numbers reflecting greater disability)], treatment lines, peripheral blood count data [ALC, absolute neutrophil count (ANC), and absolute monocyte count (AMC) at baseline (defined as the most recent blood count within 1 week before treatment initiation) and 2 weeks after treatment initiation], and the incidence and types of irAEs. In the present study, we adopted irAEs as routinely assessed by physicians. Any irAEs that occurred after 6 weeks of nivolumab administration were not counted. In addition, infusion reactions, which can be observed with the use of any monoclonal antibody, were not included as irAEs, according to previous studies (12, 25, 26).

The studies involving human participants were reviewed and approved by the ethics committees of the National Cancer Center Hospital, Keio University Hospital, and Keio University Faculty of Pharmacy, Tokyo, Japan (approval numbers: 2019-199, 20180313, and 200918-2, respectively). The study was conducted in accordance with the Declaration of Helsinki and the Ministry of Education, Culture, Sports, Science, and Technology and Ministry of Health, Labour and Welfare Ethical Guidelines for Medical and Health Research Involving Human Subjects. Japanese law does not require individual informed consent from participants in non-invasive observational trials, such as the present study. Therefore, we used the National Cancer Center Hospital and Keio University Hospital official website to provide an opt-out option rather than acquiring written or verbal informed consent from each participant.

### Endpoint

The primary endpoint was the association between peripheral blood count data (at baseline and 2 weeks after treatment initiation) and the early onset of irAEs. Consistently, with the use of potential peripheral blood biomarkers reported in previous studies (20–23), peripheral blood count data were used to calculate the ALC, NLR, and LMR. Changes in the ALC, NLR, and LMR were evaluated by comparing the 2-week values with their respective baseline values. Additionally, decrements or increments in ALC, ANC, and AMC 2 weeks after treatment initiation relative to baseline levels were evaluated. The secondary endpoint was the association of peripheral blood count data (at baseline and 2 weeks after treatment initiation) with skin reactions and diarrhea, the most frequently observed irAEs.

### Statistical Analyses

Receiver operating characteristic curve analyses and Youden’s index (27) were used to determine the optimal cut-off values of the abovementioned potential peripheral blood biomarkers to predict the early onset of irAEs. The maximum Youden’s index was calculated as sensitivity – (1 – specificity). Subsequently, positive predictive values and negative predictive values were also calculated. Multivariable logistic regression analyses were performed to assess the association between peripheral blood biomarkers and the early onset of irAEs. Potential explanatory variables concerning the patient’s background such as age, Eastern Cooperative Oncology Group performance status (2 *vs.* 0–1), and treatment line (later- *vs.* second-line treatment) were included as independent variables in the multivariable models. Shah et al. (7) reported an association between age and irAEs. Other explanatory variables were determined through clinical judgment. All statistical analyses were performed using SAS (version 9.4) and JMP (version 15.0.0; SAS Institute Inc., Cary, NC, USA). A two-sided *P-*value <0.05 was considered statistically significant.

## Results

### Patient Characteristics

**Figure 1:**
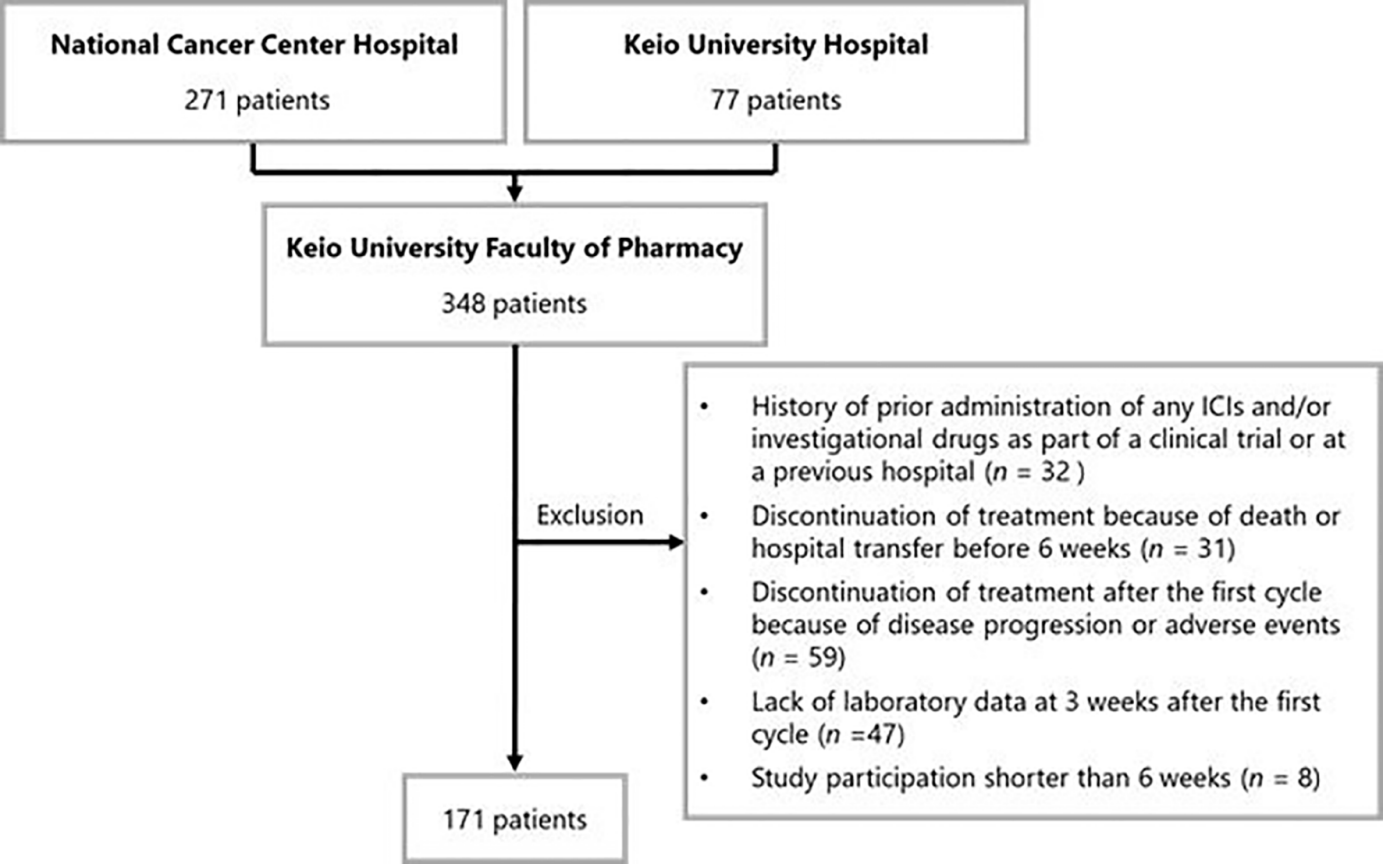
Patient enrollment flowchart. ICI, immune checkpoint inhibitor.

Of the 348 patients initially identified, 177 were excluded. Thus, 171 patients were included in the analysis. The patient inclusion flowchart is shown in **Figure 1**. Baseline patient characteristics are listed in **Table 1**. The median age of the patients was 64 [interquartile range (IQR): 56–69] years. In total, 102 (59.6%), 31 (18.1%), and 38 (22.2%) patients underwent nivolumab monotherapy as second-line, third-, and ≥fourth-line treatment, respectively. PD-L1 expression was not measured because it was not mandatory in the second- or later-line setting.

**Table 1:** Baseline clinicodemographic characteristics.

Characteristic	Patients (*N* = 171)
Age (years), median (IQR)	64 (56–69)
Sex, *N* (%)	
Male	113 (66.1)
Female	58 (33.9)
ECOG PS, *N* (%)	
0	53 (31.0)
1	101 (59.1)
2	17 (9.9)
Treatment line, *N* (%)	
Second	102 (59.6)
Third	31 (18.1)
≥Fourth	38 (22.2)

ECOG PS, Eastern Cooperative Oncology Group performance status; IQR, interquartile range.

### Endpoints

As shown in **Table 2**, 73 (42.7%) patients had early onset irAEs. Skin reactions and diarrhea were observed in 44 (25.7%) and 20 (11.7%) patients, respectively. The median time to irAEs from the initiation of nivolumab was 15 (IQR: 13–28) days. The median values for ALC, ANC, and AMC at baseline were 1,192 cells/mm^3^ (IQR: 860–1,612 cells/mm^3^), 4,688 cells/mm^3^ (IQR: 3,463–5,841 cells/mm^3^), and 449 cells/mm^3^ (IQR: 350–616 cells/mm^3^), respectively. The median values for ALC, ANC, and AMC at 2 weeks were 1,303 cells/mm^3^ (IQR: 917–1,700 cells/mm^3^), 4,590 cells/mm^3^ (IQR: 3,468–6,219 cells/mm^3^), and 459 cells/mm^3^ (IQR: 374–599 cells/mm^3^), respectively.

**Table 2:** Immune-related adverse events within 6 weeks of initiating nivolumab treatment.

Event	Patients (*N* = 171)
Any irAE, *N* (%)	73 (42.7)
Skin reaction	44 (25.7)
Diarrhea	20 (11.7)
Thyroiditis/hypothyroidism	15 (8.8)
Liver dysfunction	3 (1.8)
Pneumonitis	2 (1.2)
Encephalitis	1 (0.6)
Myasthenia gravis	1 (0.6)
Venous blood thromboembolism	1 (0.6)

irAE, immune-related adverse event.

**Figure 2:**
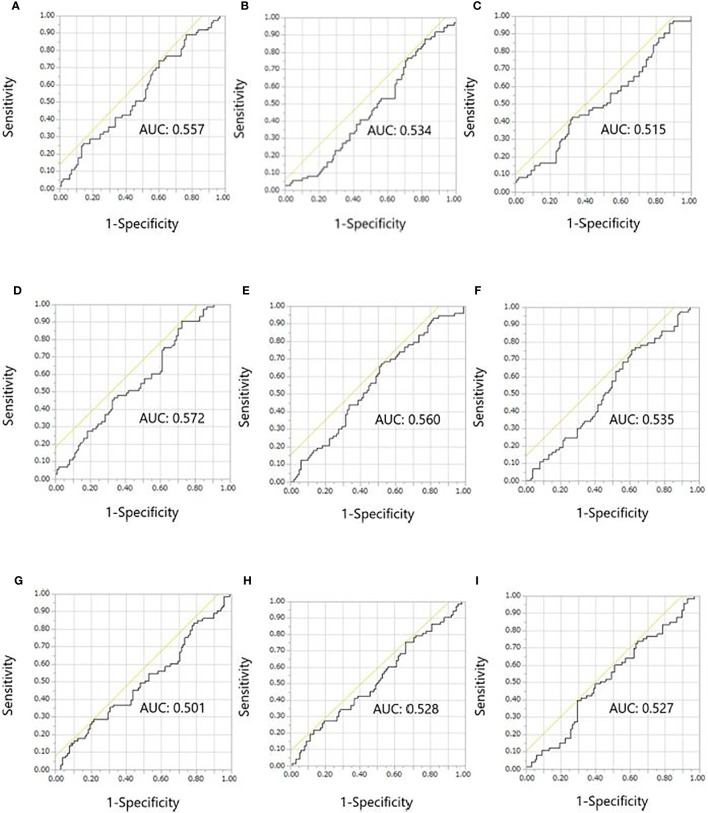
Receiver operating characteristic curves for the early onset of any immune-related adverse event. **(A)** ALC, **(B)** NLR, and **(C)** LMR at baseline. **(D)** ALC, **(E)** NLR, and **(F)** LMR at 2 weeks after treatment initiation. **(G)** ALC, **(H)** NLR, and **(I)** LMR at 2 weeks/baseline. ALC, absolute lymphocyte count; AUC, areas under the curve; LMR, lymphocyte-to-monocyte ratio; NLR, neutrophil-to-lymphocyte ratio.

Results of the receiver operating characteristic curve analyses of continuous variables are shown in **Figure 2**. Overall, the ALC, NLR, and LMR 2 weeks after treatment initiation showed relatively larger areas under the curve in the receiver operating characteristic analyses of early onset irAEs than the ALC, NLR, and LMR at baseline and changes in ALC, NLR, and LMR from baseline to 2 weeks after treatment initiation. The areas under the curve for the ALC, NLR, and LMR 2 weeks after treatment initiation were 0.572, 0.560, and 0.535, respectively. The optimal cut-off values for the ALC, NLR, and LMR were 820, 4.3, and 2.2, respectively, whereas the Youden’s index values were 0.180, 0.151, and 0.141, respectively. The sensitivity, specificity, positive predictive value, and negative predictive value for those cut-offs of ALC were 90.4, 27.6, 66.0, and 27.0%, respectively. The sensitivity, specificity, positive predictive value, and negative predictive value for those cut-offs of NLR were 67.1, 48.0, 49.0, and 47.0%, respectively. The sensitivity, specificity, positive predictive value, and negative predictive value for those cut-offs of LMR were 75.3, 38.8, 55.0, and 38.0%, respectively.

As shown in **Table 3**, multivariable logistic regression analyses revealed that ALC >820 at 2 weeks after treatment initiation was significantly associated with an increased risk of early onset of any irAE [adjusted odds ratio (OR): 3.58, 95% confidence interval (CI): 1.42–9.05; *P* = 0.007]. In contrast, no significant association was observed for NLR (>4.3; adjusted OR: 0.57, 95% CI: 0.30–1.08; *P* = 0.083) or LMR (>2.2; adjusted OR: 1.79, 95% CI: 0.90–3.56; *P* = 0.095) 2 weeks after treatment initiation. In addition, multivariable logistic regression analyses revealed that there was no significant association between decrements in ALC, ANC, and AMC from 2 weeks after treatment initiation to baseline and early onset of any irAE (adjusted OR: 1.26, 95% CI: 0.68–2.35; *P* = 0.460, adjusted OR: 1.17, 95% CI: 0.63–2.16; *P* = 0.612, and adjusted OR: 1.42, 95% CI: 0.77–2.62; *P* = 0.267, respectively).

**Table 3:** Multivariable logistic regression analyses of the early onset of immune-related adverse events.

(A) ALC at 2 weeks after treatment initiation			
Variables	Adjusted OR	95% CI	*P*-value
ALC (>820) at 2 weeks	3.58	1.42–9.05	0.007
Age (years)	1.00	0.97–1.03	0.899
ECOG PS (2)	1.07	0.35–3.29	0.908
Treatment line (later-line)	0.74	0.39–1.41	0.359

(B) NLR at 2 weeks after treatment initiation			
Variables	Adjusted OR	95% CI	*P*-value
NLR (>4.3) at 2 weeks	0.57	0.30–1.08	0.083
Age (years)	1.00	0.97–1.03	0.956
ECOG PS (2)	0.81	0.28–2.36	0.702
Treatment line (later-line)	0.74	0.39–1.40	0.357

(C) LMR at 2 weeks after treatment initiation			
Variables	Adjusted OR	95% CI	*P*-value
LMR (>2.2) at 2 weeks	1.79	0.90–3.56	0.095
Age (years)	1.00	0.97–1.03	0.989
ECOG PS (2)	0.84	0.29–2.45	0.746
Treatment line (later-line)	0.70	0.37–1.31	0.262

The multivariable logistic regression models were adjusted for age, ECOG PS (2 vs. 0–1), and treatment line (later- vs. second-line treatment).

ALC, absolute lymphocyte count; CI, confidence interval; ECOG PS, Eastern Cooperative Oncology Group performance status; LMR, lymphocyte-to-monocyte ratio; NLR, neutrophil-to-lymphocyte ratio; OR, odds ratio.

In relation to the secondary endpoint, there was no significant association of ALC >820, NLR >4.3, and LMR >2.2 at 2 weeks after treatment initiation with skin reactions and diarrhea (adjusted OR: 2.15, 95% CI: 0.85–5.45; *P* = 0.108, adjusted OR: 0.79, 95% CI: 0.41–1.54; *P* = 0.492, and adjusted OR: 1.47, 95% CI: 0.72–3.03; *P* = 0.293, respectively).

## Discussion

The peripheral blood biomarkers predictive of irAEs have been inconsistent in previous studies (8, 9) and thus, remain controversial. The present study showed that ALC >820 at 2 weeks after treatment initiation was significantly associated with an increased risk of early onset of any irAE in patients with advanced NSCLC who received nivolumab monotherapy in the second- or later-line setting. This confirms our hypothesis that peripheral blood count data can predict nivolumab-induced irAEs in patients with advanced NSCLC. To the best of our knowledge, this is the first multicenter study to investigate whether routinely available ALC can predict nivolumab-induced irAEs using a landmark analysis.

Previous studies (20–23) have focused on the association between peripheral blood count data and ICI treatment efficacy. Additionally, there have been multiple studies already published that examine the role of peripheral blood count data and the association with the development of irAEs (8, 9, 22, 28, 29). However, most of them have combined several ICI treatments such as niolumab, pembrolizumab, and atezolizumab. This could have bolstered their total numbers of patients. Nivolumab was a first in class as a second-line treatment in advanced NSCLC. Moreover, there is a difference between the treatment intervals of nivolumab (every 2 weeks) and pembrolizumab (every 3 weeks). The present study evaluated two points, at baseline and after treatment. Thus, we focused on nivolumab alone. Diehl et al. (8) reported that in patients with solid tumors who were treated with nivolumab (*N* = 125) or pembrolizumab (*N* = 42) with or without ipilimumab, ALC >2,000 at baseline or 4 weeks after treatment initiation was significantly associated with the development of grade ≥2 irAEs. Pavan et al. (9) also reported that in patients with advanced NSCLC who were treated with nivolumab (*N* = 145), pembrolizumab (*N* = 32), or atezolizumab (*N* = 7), a baseline NLR <3 and platelet-to-lymphocyte ratio <180 were significantly associated with the development of irAEs. Furthermore, our co-author previously investigated the association of pre- or post-treatment LMR and NLR values (2 and 4 weeks after nivolumab treatment) with the treatment efficacy of nivolumab and the early onset of irAEs (defined as the presentation of irAEs within 4 weeks of treatment initiation) (22). The results showed that these peripheral blood biomarkers at baseline and 2 weeks after treatment initiation were not associated with the early onset of irAEs. However, those studies did not use landmark analysis. To account for the presence of lead-time bias associated with the time-dependent development of irAEs, we conducted a 6-week analysis in accordance with previous studies (10, 11). Other reasons for choosing a 6-week period included the occurrence rate of irAEs and the timing of computed tomography. The occurrence rate of irAEs is approximately 50% within 6 weeks of initiating ICI therapy (10–14, 21). In terms of imaging, the follow-up period for patients with advanced NSCLC in Japan is generally between 6 and 8 weeks following treatment initiation (10).

The findings of the current study reveal that the ALC 2 weeks after treatment initiation may predict the early onset of irAEs in patients with advanced NSCLC and is consistent with the results of a previous study (8). In contrast, there was a discrepancy between our findings and the predictive capacity of NLR and LMR reported in some previous studies (9, 22). The reason for this discrepancy remains unclear. However, in our view, ALC is a key parameter. The interaction between anti-PD-1 and PD-L1 prevents the activation and proliferation of T cells. Inhibition of PD-L1 binding with anti-PD-1 induces T cell activation in the priming phase and increases the number of cytotoxic T cells (30). The increase in activity or number of activated T cells may result in an increased frequency of irAEs. The results of this study were consistent with our hypothesis. These findings suggest that peripheral blood biomarkers, which are evaluated after the initiation of nivolumab treatment may be useful for identifying patients at risk of early irAE onset. Importantly, the early detection of irAEs can help in reducing the negative effect on the patient’s quality of life. Our results of no association between ALC >820 and the development of skin reactions and diarrhea were underpowered (*P* = 0.108) to detect statistical significance. The number of events of skin reactions and diarrhea was 64.

The processes underlying the presentation of irAEs have not been completely clarified. Early studies (31–35) suggest several potential mechanisms, ranging from shared antigens between the tumor and the affected tissue to preexisting autoantibodies and microbiome. In this study, we primarily focused on the association of an increase in ALC and associated changes in blood count ratios with any irAE according to the potential mechanisms. These biomarkers, once validated, are easily available and do not require additional costs or setup for use in the clinical setting.

In the present study, irAEs and skin reactions were observed in 42.7 and 25.7% of patients, respectively. These incidence rates are comparable with those previously reported in the Japanese population (10–14, 19, 20, 22, 23). Overall, the incidence rate of irAEs in this study was relatively higher than that reported in other countries, and the majority of irAEs were skin reactions.

This study has some limitations. First, it was a retrospective, observational study, rather than a prospective study, and its retrospective nature does not allow formulating valid conclusions but only aids in generating a hypothesis that would require prospective validation. Patient follow-up was at the clinicians’ discretion. Thus, the possibility of information bias cannot be excluded. However, we performed multivariable analyses to reduce the effect of potential confounding factors that may be associated with observational studies and clinical differences in patient characteristics. Nevertheless, unmeasured confounders cannot be controlled during multivariate analyses because controlling these could affect the results. Moreover, we adopted irAEs, as routinely assessed by physicians. Research members retrieved the data from the electronic medical records at two hospitals. Therefore, we could not fully assess the grade of irAEs using the Common Terminology Criteria for Adverse Events (version 4.0). However, timely detection of irAEs could contribute to the proper clinical management by optimizing the treatment benefit for patients undergoing nivolumab monotherapy. Second, the sample size was relatively small despite the multicenter design. We did not conduct an additional analysis of results including only second-line treatment because the present study focused on the early onset of irAEs and not the treatment efficacy of nivolumab. Third, other types of irAEs, except those affecting the skin, rarely occurred, thereby limiting our evaluation of the types of irAEs most strongly associated with pre- or post-treatment peripheral blood biomarkers. Fourth, the complexity of the pathophysiology of irAEs is not fully understood and difficult to assess retrospectively. In a preclinical setting, specific subpopulations of lymphocytes such as CD8- and CD4-positive T lymphocytes may be associated with irAE onset (36, 37). Thus, flow cytometry analysis should be considered. Prospective efforts based on stronger scientific rationale are needed to advance in this critical field.

In conclusion, this multicenter study demonstrates that among the peripheral blood biomarkers, ALC >820 at 2 weeks after treatment initiation is significantly associated with nivolumab-induced irAEs in patients with advanced NSCLC. Clinicians should consider using ALC 2 weeks after treatment initiation for the risk stratification of patients within a 6-week study period. These findings suggest that considering the peripheral blood count data after the initiation of nivolumab monotherapy may be useful for predicting the early onset of irAEs in clinical practice. Early detection and cautious management of irAEs can optimize the treatment benefit for patients who are undergoing nivolumab monotherapy. Our data provide preliminary evidence of an association between peripheral blood biomarkers and the early onset of irAEs in Japanese patients with advanced NSCLC. These findings are likely generalizable to other Asian populations, highlighting the need for additional research in this field.

## Data Availability Statement

The raw data supporting the conclusions of this article will be made available by the authors without undue reservation. The datasets presented in this article are not readily available because of privacy and ethical restrictions.

## Ethics Statement

The studies were reviewed and approved by the ethics committees of the National Cancer Center Hospital, Keio University Hospital, and Keio University Faculty of Pharmacy, Tokyo, Japan (approval numbers: 2019-199, 20180313, and 200403-2). Written informed consent for participation was not required for this study in accordance with the national legislation and the institutional requirements.

## Author Contributions

SE and HK: conception and design. SE, HK, HH, and HN: acquisition of data (acquired the data and managed patients). SE, HK, and RU: analysis and interpretation of the data. SE and HK: writing, review, and/or revision of the manuscript. TN: designed and supervised the study. All authors contributed to manuscript revision and read and approved the submitted version.

## Conflict of Interest

RU received honoraria from Eisai, Sawai Pharmaceutical, and CAC Croit. YO received research funding from Kissei, Dainippon-Sumitomo, Ignyta, LOXO, AstraZeneca, Taiho Pharmaceutical, Chugai, Lilly, Ono Pharmaceutical, Bristol-Myers Squibb, Pfizer, MSD, Kyorin, Takeda, and Novartis and received honoraria from AstraZeneca, Taiho Pharmaceutical, Chugai, Lilly, Ono Pharmaceutical, Bristol-Myers Squibb, Pfizer, MSD, Kyorin, Takeda, Novartis, Celltrion, Amgen, and Boehringer Ingelheim. TN received research funding from Otsuka Pharmaceutical, Sanofi, Astellas Pharma, and Daiichi Sankyo.

The remaining authors declare that the research was conducted in the absence of any commercial or financial relationships that could be construed as a potential conflict of interest.

## Abbreviations

ALC, absolute lymphocyte count; AMC, absolute monocyte count; ANC, absolute neutrophil count; CI, confidence interval; ICI, immune checkpoint inhibitor; IQR, interquartile range; irAE, immune-related adverse event; LMR, lymphocyte-monocyte-ratio; NLR, neutrophil-to-lymphocyte ratio; NSCLC, non-small-cell lung cancer; OR, odds ratio; PD-L1, programmed death-ligand 1.
